# Basic Psychological Needs, Physical Self-Concept, and Physical Activity Among Adolescents: Autonomy in Focus

**DOI:** 10.3389/fpsyg.2020.00491

**Published:** 2020-03-20

**Authors:** Raúl Fraguela-Vale, Lara Varela-Garrote, Miriam Carretero-García, Eva María Peralbo-Rubio

**Affiliations:** ^1^Department of Specific Didactics and Methods of Research and Diagnosis in Education, University of A Coruña, A Coruña, Spain; ^2^GIPDAE Research Group, University of A Coruña, A Coruña, Spain

**Keywords:** basic psychological needs, physical self-concept, physical activity, autonomy, adolescence, self-determination theory

## Abstract

The contribution of this research lies in its dual approach to the question of physical activity (PA) among adolescents, combining objective measurement of PA by teenagers and a comparison of psychological satisfaction through physical activities involving differing degrees of autonomy (i.e., organized or unstructured). Using the conceptual framework of Self-Determination Theory, the analysis also examines the relationship between levels of PA among adolescents and physical self-concept and satisfaction of basic psychological needs during exercise. The study surveyed 129 first-year higher secondary education students from schools in the city of A Coruña. Satisfaction of basic psychological needs during organized and unstructured physical activities was measured using the Basic Psychological Needs in Exercise Scale. PA levels were assessed based on step count per day for a week as measured by an accelerometer. The results show that the daily step average recorded by students (7,400) is below the minimum recommended levels of PA for this age group, that students are more active on weekdays than at the weekend, and that there is no significant difference in PA levels between male and female subjects (*T* = 0.23, *p* < 0.05, *d* = 0.04). Findings from the comparative analysis of the three basic psychological needs show greater satisfaction of the need for autonomy during unstructured activities (*T* = 6.15, *p* < 0.001, *d* = 0.68), and greater satisfaction of the need for competence during organized activities (*T* = −2.50, *p* < 0.05, *d* = 0.27). No variation in terms of sex was found in relation to satisfaction of the need for autonomy or relatedness from unstructured activities; however, girls showed notably lower satisfaction than boys in relation to the need for competence (*T* = −2.62, *p* < 0.01, *d* = 0.49). Self-esteem was found to play an important mediating role and observed to be strongly related to sex (*T* = −5.16, *p* < 0.001, *d* = 0.90). Organized PA was found to provide greater need satisfaction among boys than girls across all categories. The study showed no relationship between psychological variables and objectively measured PA (Pillai’s trace: *F* = 0.86, *p* > 0.05, η^2^ = 0.08, observed power = 0.66). Basic psychological needs show significant positive interrelation between them and a significant positive relationship between them and physical self-concept, as expected based on previous literature.

## Introduction

Adolescence is a period of transition between childhood and adulthood when the influence of the family starts to fade and the peer group takes over as the main socializing force in a young person’s life (Martínez-Martínez and González-Hernández, 2017, 2018). The increasing number of decisions they begin to take autonomously transforms their daily routines and how they organize their time (Jose et al., 2011).

Numerous studies have demonstrated the importance of leisure activities during adolescence (Pronovost, 2013; Roberts, 2014; Gómez-Granell and Julià, 2015; Caballo et al., 2017), two of the core principles of which are freedom of choice and self-determination. The ability to choose how they spend their leisure time gives young people a sense of freedom, which in turn leads to greater personal development (Munné and Codina, 1996; Caldwell and Witt, 2011; Kleiber et al., 2014). Satisfying leisure experiences help to promote autonomy, formation of self-identity, social engagement and sense of achievement. Adolescence is vital time of discovery of new interests and affirmation of personal and social values and ideals (Roult et al., 2016). The transition from childhood to adolescence is a gradual process in which behaviors from both life stages coexist for a time. Of the many different leisure activities engaged in by young people, sporting and physical activity (PA) is by far the most popular (Ahedo and Macua, 2016; Fraguela-Vale et al., 2018). PA provides a range of physical, social and psychological benefits, including improved health and quality of life (Janssen and Leblanc, 2010; Ponce de León and Sanz, 2014; Vagetti et al., 2014). However, despite the numerous health benefits of PA and its high ranking among the leisure preferences expressed by young people, studies show that participation in PA during adolescence is low (e.g., Strauss et al., 2001; Barranco-Ruiz et al., 2018).

The passage from childhood to adolescence is characterized by a marked drop in PA (Abraldes and Argudo, 2009; Cairney et al., 2014; Gómez et al., 2017; Fernández-Prieto et al., 2019). To account for the sudden decline in participation at this critical point in young people’s lives, the factors associated with PA participation must be investigated and evaluated, including commonly cited variables such as sex, self-esteem, motivation and needs satisfaction (Martín-Matillas et al., 2011; Ponce de León and Sanz, 2014).

A number of studies on PA use Self-Determination Theory (SDT) to analyze motivation and psychological processes that underlie well-being (Deci and Ryan, 1985, 2000, 2002; Moreno et al., 2008a, b; Ntoumanis and Standage, 2009; Franco et al., 2017). SDT is a macro-theory of motivation and personality that can be applied to different contexts and cultures. As explained by Stover et al. (2017), SDT is based on the assumption that people have a natural tendency toward growth, and actively seek to manage their environment and interactions and integrate new experiences into their sense of self. Satisfaction of their needs and their motivation to carry out or engage in different activities is therefore subject to a combination of internal, external and contextual factors. SDT comprises six mini-theories, which correspond to different aspects of the relationship between motivation and emotion, behavior and well-being (Deci and Ryan, 2008; Krause et al., 2019). Causality Orientations Theory (COT) describes the different orientations in people’s personalities that affect their response to environmental stimuli, and how this impacts on their capacity to make decisions and regulate their behavior (Moreno and Martínez, 2006; Stover et al., 2017). Cognitive Evaluation Theory (CET) specifies factors associated with variability in intrinsic motivation, based on the assumption that innate interests may vary depending on environmental factors and their psychological effect on the individual (Moreno and Martínez, 2006; Stover et al., 2017). Organismic Integration Theory (OIT) details the different forms of extrinsic motivation, and the contextual or social forces which may support or impede the individual’s capacity to internalize and integrate the regulation of these behaviors (Ryan and Deci, 2000; Stover et al., 2017). Goal Contents Theory (GCT) distinguishes between extrinsic and intrinsic goals and examines their effect on behavior and well-being. While extrinsic goals focus on interpersonal comparison and other people’s reactions, intrinsic goals are more associated with satisfaction of basic psychological needs and greater psychological well-being. Relationships Motivation Theory (RMT) broadens the scope of relatedness as a basic psychological need, explaining that satisfaction of the need for relatedness is determined by the quality of the relationship and the mutuality of perceived autonomy and autonomy support between partners (Deci and Ryan, 2014).

The last of these mini-theories, and the one on which this study is based, is Basic Psychological Needs Theory (BPNT), which postulates competence, autonomy and relatedness as innate, universal and essential needs upon whose satisfaction our health and well-being depend. Competence refers to the ability to perform tasks of varying complexity, and is positively associated with the ability to regulate stress, self-esteem and well-being; low competence is associated with depression, anxiety and low self-esteem (Parfitt et al., 2009). Autonomy refers to the feeling that the locus of causality for the things one does is internal: that one’s actions are one’s own and not due to external factors (Ryan and Deci, 2007). Perception of autonomy is a positive predictor of satisfaction of both competence and relatedness, which in turn is a positive predictor of self-determined motivation (Castillo et al., 2002; Standage et al., 2006). Relatedness is the extent to which a person feels securely part of a group or connected with others within his or her social context. Basic psychological needs are considered necessary to mediate intrinsic and extrinsic motivation and demotivation.

Basic Psychological Needs Theory provides the theoretical framework for the research instruments used for this study, some of which are focused specifically on PA. The study used the Spanish version (Sánchez and Núñez, 2007) of the Basic Psychological Needs in Exercise Scale (BPNES) (Vlachopoulos and Michailidou, 2006). According to BPNT, respondents who report satisfaction in relation to the three dimensions tested by the scale (autonomy, competence, and relatedness) show higher levels of intrinsic motivation and therefore greater self-determination, which in turn leads to greater participation in PA and improved health (Almagro et al., 2011; Sánchez-Oliva et al., 2013; Sicilia-Camacho et al., 2014; Morillo et al., 2018). Nevertheless, scores obtained from BPNES testing are dependent on a wide array of variables, including sex, age, type of activity, and how the activity is organized (Lamoneda and Huertas-Delgado, 2019). In relation to sex, while some studies report a tendency for boys to score higher than girls across all three dimensions (Brunet and Sabiston, 2009), others show variation between results only in relation to perceived autonomy and competence (Gómez-Rijo et al., 2014). Sex considerations aside, these studies all highlight the importance of BPNES values as predictors of participation in PA.

One important line of research in Spain has been the adaptation of BPNES to Physical Education teaching in schools in an attempt to encourage participation in PA outside of school by increasing satisfaction of psychological needs (Méndez-Giménez et al., 2015). A recent study by Pineda-Espejel et al. (2019) has developed a Spanish version of the scale adapted to sports. In research involving adults, analysis of variables such as level of organization show that participants in more structured activities (Hellín et al., 2006) or federated sports (Edmunds et al., 2007) display higher levels of perceived competence. One of the central research questions of this study is to compare satisfaction of basic psychological needs through unstructured PA with that achieved through more organized, often institutionalized PA.

Research has also shown a link between competence, autonomy, and relatedness with engagement in PA by teenagers. Babic et al. (2014) report a direct association between higher levels of PA and higher values of perceived competence in physical exercise, fitness, appearance, and physical self-concept. There is little information, however, regarding the directionality of this relationship, which may be one of interdependence in certain cases. It is also unclear whether PA has a positive influence on physical self-concept, or if people with higher perception of their motor competence are more likely to engage in PA (De la Torre-Cruz et al., 2018). The question of autonomy is linked to the gap in biological maturity that emerges in adolescent girls and boys of a similar chronological age (Cairney et al., 2014). The fact that girls mature earlier than boys means that they also begin to make more autonomous decisions about their leisure time at a younger age, leading to differences between the two in their leisure time and PA choices. This contrast is caused not only by the different preferences of boys and girls, therefore, but also by the latter’s earlier cognizance of the leisure habits and routines of adult life. Teenage girls tend to opt for non-structured types of PA, while their male peers continue to take part in more organized, often competitive forms of PA (García-Moya et al., 2012; Castro-Sánchez et al., 2016; Fraguela-Vale et al., 2020). Finally, the need for relatedness is a strong inducement to engage in physical exercise owing the significant positive psychological effect it has on those who do. The perception of relatedness is associated with a sense of connection and acceptance by others, and its satisfaction leads to greater well-being, security and unity between members of the community (Sánchez and Núñez, 2007), as well as a greater propensity to participate in PA (Peres et al., 2012). In fact, certain studies have found that emotional bonds with family members and peers are an important factor in young people’s self-determined motivation to participate in PA and thereby strengthen their emotional ties and build up social support networks (Balaguer et al., 2008; Cheng et al., 2014).

The present study also assessed physical self-concept, as one of the main mediators between psychological variables (in this case, basic psychological needs) and participation in PA. Franco et al. (2017) highlight the influence of physical self-concept on the intention to engage in PA, observing that teenagers with higher self-concept are more likely to continue to engage in daily PA in the future. Physical self-concept is influenced by subjects’ self-perception of their physical appearance, level of fitness, sporting ability and strength (Goñi et al., 2004), with the highest scores in relation to self-concept and PA recorded among subjects who have been engaged in PA for longer (Reigal et al., 2012; Reigal-Garrido et al., 2014; López-Cazorla et al., 2015; Kyle et al., 2016). Research focusing on teenagers reveals that the relationship between PA and physical self-concept in adolescence is also significant, with factors such as age and sex playing decisive roles in this association (Fernández-Bustos et al., 2019).

In most of the studies cited thus far as evidence of the relationship between PA and psychological variables (basic psychological needs, physical self-concept, motivation, etc.), PA levels are measured indirectly using self-reporting instruments completed by subjects. The results in relation to PA participation in these studies show a clear relationship between PA and psychological variables, in keeping with the postulates of BPNT. More recently, however, studies in Self-Determination Theory and its mini-theories have begun to use objective measurement of PA, the findings of which reveal a much less clear-cut relationship between PA and psychological variables. In their review of research into the relationship between PA and self-concept, for example, Babic et al. (2014) advise caution based on their finding that 84% of the studies analyzed contained a high risk of bias owing to the use of self-reporting tools. As well as creating the possibility of bias (social desirability and cognitive difficulty), the use of self-reporting for both PA and physical self-concept also produces higher correlations between the two. The few studies which do use objectively measured PA data focus on the relationship between PA and self-esteem, and show contrasting results: while some find little or no solid evidence of a relationship between psychological variables and exercise (Raustorp et al., 2005; Cuddihy et al., 2006), others report a strong link between objectively measured PA and self-perception (Morgan et al., 2008).

For this study, therefore, it was judged particularly important to include objective measurement of PA and to compare these data with the psychological variables of BPNT. Recent research has identified step counting as a valid, objective standard of measurement of daily PA, particularly in the light of studies by leading authors in this area, such as Tudor-Locke and her colleagues. Since the publication of their review of the existing literature to determine the minimum number of steps needed by children and adolescents to be equivalent to 60 min of moderate to vigorous PA (Tudor-Locke et al., 2011), advances in wearable technologies have given us highly accurate measuring devices that can be worn during almost any activity (Hibbing et al., 2016). For this reason, more and more authors are now adopting objective step counting as a method of quantifying PA in research ranging from PA promotion programs for young children (Cameron et al., 2016) to the categorization of adolescents as active or inactive (Benítez-Porres et al., 2016).

The significance of this study lies in its dual approach to the question of PA among adolescents, which combines objective measurement of PA by teenagers, and a comparison of psychological satisfaction levels achieved from PA based on the degree of autonomy involved (organized or unstructured) during a period of transition toward less structured exercise. In addition, the study also examines to what extent physical self-concept among adolescents and satisfaction of their basic psychological needs through exercise are affected by their level of participation in PA. Based on the evidence presented in this introduction, we propose the following hypotheses: (1) that the level of participation in PA by adolescents is lower than the recommended amount of healthy daily exercise; (2) that satisfaction of the need for autonomy is greater when achieved through unstructured PA, while the effect of exercise on the need for competence and the need for relatedness is the same whether organized or unstructured; and (3) that objectively measured PA levels have little or no influence on the satisfaction of basic psychological needs through exercise.

## Methodology

### Participants

The study sample was selected using non-probability sampling based on the criterion of convenience of accessibility. The study involved 129 first-year higher secondary education students from three schools in the city of A Coruña. The sample comprised participants with an average age of 16.07 years (*SD* = 0.70), 66 girls (51.2%) and 63 boys (48.8%), from two state schools (72.9%) and one state-funded private school (27.1%). The proportion of students in each of the three study tracks was as follows: Science and Technology, 37.2%; Humanities and Social Sciences, 60.5%; Arts, 2.3%.

### Measurement Instruments

The research was carried out using the following instruments:

#### Basic Psychological Needs in Exercise Scale (BPNES)

The study used the Spanish version (Sánchez and Núñez, 2007) of the Basic Psychological Needs in Exercise Scale (BPNES) (Vlachopoulos and Michailidou, 2006). The instrument comprises twelve items, divided into three dimensions (four items per dimension): autonomy (“The exercise program I follow is compatible with my choices and interests”; “The way I exercise matches perfectly the way I like to exercise”; “The way I exercise meets my desires”; “I get to choose how I exercise”); competence (“I feel I have made great progress toward achieving my goals”; “I do the exercises effectively”; “Exercise is an activity I do very well”; “I feel that I can meet the requirements of my training program”); and relatedness (“I feel very comfortable when I do exercise with the rest of the exercise participants”; “I have a very friendly relationship with the other exercise participants”; “I feel that I can communicate freely with the other exercise participants”; “I feel very comfortable with the other exercise participants”). The introductory phrase for each item is “When I do exercise…” Responses were scored using a Likert-type scale, ranging from 1 (strongly disagree) to 5 (strongly agree). The alpha reliability coefficients obtained were 0.74 for autonomy, 0.87 for competence, and 0.81 for relatedness.

In order to measure the satisfaction of psychological needs through PA carried out with differing degrees of autonomy, participants were asked to complete two scales: one for unstructured exercise, and one for more organized exercise (usually institutional and adult-led). Respondents without participation in one or either of the two types of activity were instructed to leave the corresponding scale or scales blank. All participants were advised that the subject of Physical Education should be not be taken into account in their assessments, as Physical Education is a compulsory form of PA. The Cronbach’s alphas obtained for each of the three dimensions in relation to unstructured exercise were as follows: autonomy, 0.74; competence, 0.76; and relatedness, 0.90. Reliability coefficients in relation to organized exercise were: autonomy, 0.63; competence, 0.77; and relatedness, 0.85.

#### Physical Self-Concept

Physical self-concept was measured using the corresponding items from García and Musitu’s (2014) AF5: Self-concept form 5 (Autoconcepto Forma 5), which measures academic, social, emotional, family, and physical self-concept. The scale comprises thirty items, six per dimension, with a response scale ranging from 1 to 99. AF5 is one of the most widely used measuring instruments for self-concept among Spanish-speaking samples (e.g., Lila et al., 2000; Pellerano et al., 2006; Méndez-Giménez et al., 2013; Martínez-Martínez and González-Hernández, 2017, 2018; Onetti-Onetti et al., 2019). The structure of the scale has been empirically tested using exploratory factorial analysis (García and Musitu, 2014) and confirmatory factorial analysis (García et al., 2006, 2011). Cronbach’s alpha coefficient for physical self-concept is reported in these studies as 0.73 in the Spanish context.

For this study, participants were asked to answer only the six items corresponding to physical self-concept: “I’m good at sports”; “I like myself physically”; “I’m attractive”; “People look for me to do sports activities”; “I look after myself physically”; and “I consider myself elegant.” Cronbach’s alpha coefficient for physical self-concept was 0.79.

#### Physical Activity (PA) Levels

The level of daily PA among students over the course of a week was measured using a wristband with a built-in ADXL362 three-axis accelerometer. Finding from previous studies have demonstrated the precision and reliability of accelerometers as a method of measuring objective PA (Ling et al., 2015; Eyre et al., 2016; Alvis-Chirinos et al., 2017). During the first hours of wearing, users can often be very conscious of the wristband device, the novelty value of which may act as a temporary motivation to engage in PA. To minimize the impact of this response, data from the 1st day of wearing were not included in the analysis. The same exclusion was applied to the day the wristbands were collected, on the grounds that the data for that day were incomplete. Subjects’ levels of PA were thus measured for a full week, not counting these discounted days. Where researchers were advised by participants that their accelerometers were not counting their steps properly and the problem of miscounted steps was confirmed, all affected data were eliminated from the analysis. Participants were requested to indicate on the questionnaire if they had forgotten to activate their accelerometer for any significant period of time (more than 3 h), so that data from that day could be omitted from the total activity score. Atypical daily step values (less than 350, more than 35,000) were isolated and also removed from the analysis.

To avoid the impact of seasonal variation on PA levels (Carson and Spence, 2010; Rahman et al., 2019), all PA measurements were carried out at the same time of year (autumn 2019). Weather conditions can also have a significant influence on PA levels (Rahman et al., 2019), so provision was made for the elimination of data recorded on days when conditions were especially adverse (e.g., intense rain or cold). However, no data were required to be removed on these grounds.

In addition to calculating the average number of steps taken by participants, the analysis also grouped results according to the index categories established by Tudor-Locke and Bassett (2004): “sedentary” (<5,000 steps per day), “low active” (5,000–7,499 steps), “somewhat active” (7,500–9,999), “active” (10,000–12,499), and “highly active” (>12,500). In view of the low number of participants in the final two ranges, these scores were classed together as “active or highly active.”

#### Number of Physical Activities Carried Out

Participants were asked to state the different physical activities performed over the course of the week. Spaces were provided in the questionnaire for two unstructured activities and two organized activities on weekdays, and two unstructured activities and two organized activities on the weekend. The range of values for each type of activity was 0–4, with a maximum total value of 8 as the sum of the week’s activities.

#### Level of Autonomy in Physical Activities Carried Out

Level of autonomy was calculated based on the number of activities carried out. Participants who practiced just one activity were classified as “unstructured” or “organized” depending on the type of activity performed. Where the difference between activity types was more than 1, participants were classified according to the higher scoring activity. Where the difference between activity types was less than or equal to 1, participants were classified as “mixed” (both unstructured and organized).

### Procedure

The principals of the schools selected for the study were contacted and informed of the objectives and procedure of the study. Once the schools’ agreement had been obtained, permission for students to participate in the study was requested from their families using an informed consent form, to be taken home, signed by parents or guardians, and returned (if favorable).

Following selection of the group of participants from each establishment, two members of the research team were sent out to the schools to fit participants one-by-one with their accelerometer (i.e., activity wristband), and distribute and collect the questionnaire. The whole process was overseen by the group’s Physical Education teacher and the two research team members, who also dealt with any questions the students might have. Eight days after this first encounter, one member of the research team returned to the school to collect the wristbands.

### Data Analysis

Data were analyzed using IBM SPSS Statistics V25 (Armonk, NY, United States) and G^∗^Power 3.1 (Faul et al., 2007).

Descriptive analysis was used to ascertain the number of steps per day, the level of satisfaction of basic psychological needs in unstructured and organized activities, and participants’ physical self-concept.

Since different scales were used to measure dependent and independent variables, the data were normalized by converting scale scores into standardized *Z* scores. In view of the variable nature of the dependent variable (daily PA by adolescents of different ages), a Kolmogorov–Smirnov test was run to determine the normality of the sample. The results confirm that the sample shows a normal distribution of values for the variables “total weekly steps,” “weekday steps,” and “weekend steps” (*p* > 0.05) and that parametric testing is therefore possible.

Inferential analysis was used to confirm the existence of linear correlation between psychological variables (basic psychological needs and physical self-concept) and objectively measured PA. To compare the difference between satisfaction of basic psychological needs through unstructured PA and through more organized PA, a Paired Samples *t*-Test was applied. To study the influence of sex on psychological variables and PA, a Independent Samples *t*-Test was applied. In both instances, effect size was calculated using G^∗^Power 3.1 based on power (1−β), α = 0.05 and two queues, to quantify the magnitude of the difference between the two averages and the *post hoc* statistical power of the tests. A chi-squared test was used to examine the relationship between level of autonomy in PA and sex; a Mann–Whitney *U* test was run to study the effect of sex on the number of weekly physical activities performed, and a Wilcoxon signed-rank test was carried out to compare the number of unstructured and organized activities recorded.

To assess the influence of PA on perceived satisfaction of basic psychological needs and physical concept, a multivariate analysis of variance (MANOVA) was carried out using the psychological variables as dependent variables, and the summary measure of steps taken by adolescents over the course of a week as an independent variable. As advanced in the introduction, the sex of participants was analyzed as a co-variable owing to its possible influence on the relationship between PA and psychological variables.

## Results

### Physical Activity

The results (Table 1) show that the average number of steps taken by students per week is around 7,000. Students are more active on days when they have classes than on the weekend. As expected, a high correlation was found between the weekly step count average and the daily step count averages for weekdays and the weekend. However, the study also showed a correlation between weekday values and weekend values that suggests the existence of a pattern of behavior in relation to PA according to which very active subjects remain very active throughout the week, and very inactive subjects maintain consistently high levels of inactivity.

**TABLE 1:** Mean ± SD, asymmetry and correlation between total weekly, weekday, and weekend steps.

PA (steps)	1	2	3	*M*	*SD*	As	*K*
(1) Weekly	–			7428.08	3197.22	1.13	1.49
(2) Weekday	0.96***	–		8295.71	3585.12	1.30	2.44
(3) Weekend	0.71***	0.49**	–	5187.05	3375.77	0.61	−0.22

**p < 0.01, ***p < 0.001; M = mean; SD = standard deviation; As = asymmetry; K = kurtosis.

Based on the indexing criteria for weekly step counts set out in the methodology section, the distribution of subjects by step index category is as follows: 23.1% sedentary, 37.6% low active, 23.1% somewhat active, and 16.2% active or very active.

Regarding the number of activities performed, the average for unstructured activities was 2.57 (*SD* = 1.31) out of a possible 4, indicating that participation in this type of PA extends to both weekdays and weekends. The average number of organized activities was 1.46 (*SD* = 1.30). The Wilcoxon signed-rank test shows a highly significant difference between participation in each type of activity (*Z* = −6.05, *p* < 0.001). The adolescents surveyed in this study thus present a clear preference for unstructured activities over institutionalized activities. The overall average number of activities (both structured and unstructured) was 3.99 (*SD* = 1.95) out of a possible 8.

A similar distribution of values was obtained among subjects at the more autonomous end of the scale, with 48.8% classed as unstructured only and 43.9% as mixed. Only 7.3% of the sample reported participating in organized PA only.

The comparison of PA levels according to sex (Table 2) reveals a largely homogeneous group, with little significant difference between average step counts for male and female subjects, either on weekdays or at the weekend. It is important to note, however, the high dispersion of the dataset, as denoted by the very high standard deviation across all parameters.

**TABLE 2:** Physical activity (PA) comparison between male and female subjects.

PA (steps)	Sex	*N*	*M* (*SD*)	*T*	*d*	*P*
Weekly	Female	60	7494.09 (3268.83)	0.23	0.04	0.07
	Male	57	7358.60 (3147.58)			
Weekdays	Female	60	8301.11 (3721.95)	0.02	0.00	0.05
	Male	57	8290.00 (3468.30)			
Weekend	Female	57	5551.72 (3409.37)	1.21	0.23	0.32
	Male	48	4753.99 (3318.72)			

*M, mean; SD, standard deviation; T, T-values (Independent Samples t-test); d, Cohen’s d; P, observed power.*

Regarding the number of activities carried out by each sex (Mann–Whitney *U* test: *Z* = −4.36, *p* < 0.001), however, differences in terms of sex were more pronounced, with female subjects reporting an average of 3.29 (*SD* = 1.88) activities per week, compared to 4.73 for their male counterparts (*SD* = 1.75).

A chi-squared test was used to assess the relationship between sex and level of autonomy in PA. In view of the low number of respondents in the “organized PA” group, these subjects were grouped together with the “mixed” participants to permit a comparison between subjects whose PA habits include unstructured activities only and subjects whose PA habits include some organized activities. The results reveal the influence of sex on autonomy of PA (chi-squared = 5.94, *p* < 0.05, *w* = 0.26), with girls tending to be more autonomous in their practice and boys more likely to opt for organized activities only or a combination of both types.

The study thus reveals a distinct pattern of PA practice among girls, consisting of fewer and less varied (mostly unstructured) types of activities, yet similar overall levels of PA participation across both sexes.

### Basic Psychological Needs and Autonomy in PA

As Table 3 shows, satisfaction of basic psychological needs through exercise (organized or unstructured) is consistently high, while results for physical self-concept show values of just 60 out of a maximum 99.

**TABLE 3:** Satisfaction of basic psychological needs in unstructured and organized activities, physical self-concept, and weekly PA.

	*M*	*SD*	As	*K*
Autonomy (U)	4.13	0.77	−1.10	1.99
Competence (U)	3.80	0.82	−0.74	−0.81
Relatedness (U)	4.38	0.85	−1.86	3.29
Autonomy (O)	3.62	0.71	−0.36	−0.08
Competence (O)	4.04	0.76	−0.63	−0.42
Relatedness (O)	4.40	0.78	−1.38	1.05
Physical self-concept	60.27	20.06	−0.49	−0.28

*U, unstructured PA; O, organized PA; M, mean; SD, standard deviation; As, asymmetry; K, kurtosis.*

As expected, satisfaction of the need for autonomy is significantly higher in unstructured activities than in organized ones (Table 4), and effect size is high (Cohen, 1988). Differences are also observed in relation to satisfaction of the need for competence, with organized activities appearing to satisfy the need for competence more than unstructured activities. The significance of this difference should be interpreted with caution, however, as effect size in this case is small.

**TABLE 4:** Satisfaction of basic psychological needs for unstructured and organized PA.

	PA	*N*	*M (SD)*	*T*	*d*	*P*
Autonomy	Unstructured	83	4.16 (0.72)	6.15***	0.68	0.99
	Organized		3.61 (0.71)			
Competence	Unstructured	83	3.85 (0.81)	−2.50*	0.27	0.68
	Organized		4.04 (0.76)			
Relatedness	Unstructured	81	4.38 (0.82)	0.30	0.03	0.05
	Organized		4.36 (0.81)			

*p < 0.05, ***p < 0.001; M, mean; SD, standard deviation; T, T-values (Paired Samples t-test); d, Cohen’s d; P, observed power.

No significant differences are observed in relation to the effect of activity type on satisfaction of the need for relatedness.

Table 5 summarizes the results of the comparison of male and female subjects in relation to all the psychological variables discussed above. Values for satisfaction of the need for autonomy and relatedness through unstructured activities are similar for both groups of subjects. For all the other parameters, the results show higher levels of satisfaction of psychological needs for male subjects than for their female counterparts. Results for physical self-concept also show significant variation between male and female participants, with values for the former scoring close to 70 points out of a possible 99, and the latter averaging just over 50. This is the only case in which a large effect size is observed.

**TABLE 5:** Satisfaction of basic psychological needs and self-concept for male and female students.

	Sex	*N*	*M (SD)*	*T*	*d*	*P*
Autonomy (U)	Female	61	4.02 (0.86)	−1.42	0.26	0.41
	Male	59	4.22 (0.65)			
Competence (U)	Female	61	3.60 (0.84)	−2.62**	0.49	0.85
	Male	59	3.99 (0.74)			
Relatedness (U)	Female	57	4.23 (1.02)	−1.89	0.35	0.59
	Male	58	4.53 (0.62)			
Autonomy (O)	Female	38	3.39 (0.85)	−2.50**	0.55	0.81
	Male	51	3.78 (0.53)			
Competence (O)	Female	38	3.84 (0.88)	−2.02*	0.44	0.66
	Male	51	4.18 (0.61)			
Relatedness (O)	Female	38	4.17 (0.99)	−2.20*	0.49	0.73
	Male	51	4.56 (0.52)			
Physical self-concept	Female	66	52.19 (21.37)	−5.16***	0.90	0.99
	Male	63	68.71 (14.49)			

*p < 0.05, **p < 0.01, ***p < 0.001; U, unstructured PA; O, organized PA; M, mean; SD, standard deviation; T, T-values (Independent Samples t-test); d, Cohen’s d; P, observed power.

### Basic Psychological Needs, Physical Self-Concept and PA

The different parameters of satisfaction of basic psychological needs show significant positive interrelation between them and a significant positive relationship between them and physical self-concept (Table 6). None of the psychological variables (basic psychological needs and physical self-concept) show a correlation with PA (average steps per week). High correlations (>0.50) are observed between the three dimensions tested by the BPNES in relation to both organized and unstructured PA. Competence and relatedness show a high correlation (>0.60) in relation to both types of activity. However, the same is not true of autonomy, the correlation for which, though still significant, is only 0.37.

**TABLE 6:** Correlation analysis between the variables.

	1	2	3	4	5	6	7	8
(1) A (U)	–							
(2) C (U)	0.61***	–						
(3) R (U)	0.60***	0.55***	–					
(4) A (O)	0.37***	0.37***	0.31**	–				
(5) C (O)	0.41***	0.60***	0.50***	0.68***	–			
(6) R (O)	0.32**	0.35***	0.67***	0.52***	0.63***	–		
(7) PSC	0.24**	0.50***	0.33***	0.50***	0.62***	0.46***	–	
(8) PA	0.04	0.11	−0.03	−0.06	0.03	−0.07	0.12	–

**p < 0.01, ***p < 0.001; A, autonomy; C, competence; R, relatedness; PSC, physical self-concept; U, unstructured PA; O, organized PA.

To determine the influence of PA on psychological variables, a multivariate analysis of variance (MANOVA) was carried out. Box’s *M* test shows that the covariance matrices of the dependent variables are equal across groups (*p* < 0.05). Levene’s test of equality of error variances shows that error variances are equal across groups (*p* > 0.05), except in relation to autonomy for unstructured PA (*p* < 0.05).

The MANOVA found that the interaction of dependent (psychological) variables was not affected by PA levels (Pillai’s trace: *F* = 0.86, *p* > 0.05, η^2^ = 0.08, observed power = 0.66). In contrast, the sex of participants, analyzed here as a co-variable, was found to have a significant principal effect (Pillai’s trace: *F* = 2.93, *p* < 0.01, η^2^ = 0.24, observed power = 0.90).

The paired comparison shows variation due to sex in relation to satisfaction of competence through unstructured PA among sedentary and low/somewhat active subjects (*p* < 0.05).

Results from a multiple regression analysis conducted are not displayed here since the analysis yielded no significant results owing to the lack of correlation between PA and the different parameters of satisfaction of basic psychological needs and physical self-concept.

## Discussion

One of the main objectives of this accelerometry-based research has been to “translate” the daily step targets recommended by the World Health Organization (World Health Organization [WHO], 2010) for children and teenagers, and to determine the minimum number of steps needed to be considered “active.” In Spain, Parra et al. (2018) propose a threshold of approximately 10,000 steps per week for adolescents (11,000 for boys and 10,500 for girls), yet research shows that the tendency is actually for PA levels to decline significantly during the transition between primary and secondary school (Lau et al., 2017). Step averages recorded by the adolescent participants in our research were found to be below the minimum recommended levels of PA for this age group, thus confirming the first hypothesis of our study. Research in this area suggests that this finding is in keeping with the prevailing trend in other developed countries (Cameron et al., 2016).

Results from other studies also highlight significant quantitative variability between weekday and weekend PA among adolescents (Pelclová et al., 2010; Manzano-Sánchez and Valero-Valenzuela, 2018). Nor is the phenomenon confined to teenagers: Czajka et al. (2015) have observed a similar trend in childhood, which they attribute to the influence of the children’s parents. In the case of adolescents, other factors, such as access to night-time leisure activities, may also affect their weekend levels of PA. Adolescents are therefore more active on school days and less likely to engage in PA at the weekend, when, conversely, they have more free time available.

The study showed little variation between girls and boys in relation to step count. These results are in line with the findings of authors such as Tudor-Locke et al. (2011), whose often-cited review of research into PA and step count among different age groups finds variation between step indices for male and female subjects to be lowest among pre-school and adolescent populations. In contradistinction to this view, numerous other studies report variation in step count results for male and female adolescents (Adams et al., 2013; Barreira et al., 2015; Hubáčková et al., 2016).

Although girls took part in fewer activities during the week, their overall PA levels were comparable to those of their male peers. Despite the distinct participation profile exhibited by each group (males and females), their overall amount of PA (number of steps) was similar. The difference between them may be attributed to two factors. The first factor refers to the traditional preference among girls for individual, unstructured and less competitive physical activities, in contrast to the male preference for more organized, collective, competitive PA. The second factor relates to the different experience of adolescence by girls and boys: whereas girls at this age appear to be developing more individual, independent, adult-like attitudes to PA, boys show more prolonged involvement in the sporting and PA routines of their childhood and less progress toward more adult forms of physical leisure. In this way, boys’ routines show a more even balance between organized and unstructured activities, while girls display a clear preference for more self-managed, non-institutionalized PA.

In order to establish which basic psychological needs are satisfied by organized PA and which by unstructured PA, the variables were analyzed separately in relation to each type of activity. As predicted by the second hypothesis of this research, the comparative analysis shows highly significant differences in relation to satisfaction of the need for autonomy through unstructured activities. This result is especially significant considering that the comparison applied only to participants with involvement in both organized and unstructured activities, not to participants whose PA habits include unstructured activities only. The findings from this mixed group show a higher level of satisfaction of the need for autonomy from unstructured activities. The second hypothesis has proved only partially accurate, however: in contrast to the assumption that both types of activity would affect satisfaction of the remaining two basic psychological needs equally, the results of the study show greater satisfaction of the need for competence from organized activities. The effect size for this difference is small, however, so its significance should be interpreted with caution.

The results in relation to sex confirm the idea that boys tend to score higher across all three dimensions of the scale (Brunet and Sabiston, 2009; Gómez-Rijo et al., 2014). In this study, organized PA was found to provide greater need satisfaction among boys than girls in all categories. In unstructured activities, boys and girls showed similar satisfaction levels in relation to the need for autonomy and relatedness, but girls showed notably lower scores in relation to the need for competence. Self-esteem was found to play an important mediating role and observed to be strongly related to sex: the results revealed that both physical self-concept (Malo et al., 2011) and satisfaction of the need for competence (through both organized and unstructured activities) were significantly lower among female subjects. Lower physical self-concept may account, at least in part, for the difficulty experienced by girls in finding forms of PA that satisfy their need for competence. Our results are consistent with the findings of Lamoneda and Huertas-Delgado (2019), who recommend the implementation of measures to satisfy the need for competence as a strategy to increase levels of PA among adolescents, especially girls. The question of exercise intensity is another possible factor to explain the differences observed between the two sexes in relation to satisfaction of basic psychological needs and physical self-concept. Girls tend to participate in fewer and more unstructured activities, which are in turn of a generally low intensity (e.g., walking). Boys, on the other hand, participate in more activities, frequently of an organized nature and a higher intensity. It may be that higher intensity activity has a greater positive effect on self-concept and satisfaction of basic psychological needs than its lower intensity equivalents (Candel et al., 2008; Parfitt et al., 2009; Goñi and Infante, 2010).

Much of the research on the relationship between PA and the psychological variables of BPNT measure PA indirectly using data from self-reporting instruments. The emergence in recent years of a growing number of resources for measuring PA objectively has opened up a new line of research in this area. While data from comparative studies of psychological variables and objectively measured PA are still sparse, the findings so far are inconclusive and even contradictory.

The Basic Psychological Needs mini-theory of Self-Determination Theory postulates a positive relationship between satisfaction of the three basic psychological needs (autonomy, competence, and relatedness) and greater intrinsic motivation to engage in PA, leading to greater self-esteem and a higher level of participation in PA (Franco et al., 2017). The mini-theory is based on the general assumption that satisfaction of basic psychological needs and higher intrinsic motivation make young people more likely to participate in healthful physical activity (Almagro et al., 2011; Sánchez-Oliva et al., 2013; Sicilia-Camacho et al., 2014). Our data, however, show no such relationship between psychological variables and objectively measured PA, as predicted by our third hypothesis. The three dimensions tested by the BPNES are positively interrelated and show a positive relationship with physical self-concept, as predicted by BPNT, but there is no corresponding correlation with the different levels of PA recorded in the study. This outcome is in keeping with the findings of other studies using objective measurement instruments to quantify daily PA, where no significant relationship is observed between steps per day and psychological variables (Raustorp et al., 2006, 2009; Raustorp and Lindwall, 2014).

The absence of a clear relationship between psychological variables and objectively measured PA is not surprising, given that objectively measured daily step counts take into account activities not traditionally reported as PA. Everyday activities such as walking the dog (Wohlfarth et al., 2013) or walking to and from school (Baig et al., 2009) can make a significant difference to recorded PA, especially among less active groups, and even more so when repeated over the course of several days. As regards the possible limitations or inaccuracy of objectively measured PA, it is important to point out that pedometers and accelerometers tend to underestimate levels of PA achieved through sports and are unable to measure activity intensity, which in the case of organized PA tends to be higher than that associated with everyday activities such as walking. As people age and organized sports activities assume less prominence in their daily routines, step counting becomes a truer reflection of the amount and pace of PA. To increase the accuracy of our instruments, the relationship between psychological variables and PA must be reinterpreted from a new perspective that takes into account the concept of everyday PA. When participants are surveyed regarding their perceptions about physical activity, a significant correlation between psychological variables and physical exercise is observed. However, data relating to the actual physical activity performed may see some subjects classed as less active, despite engaging in levels of activity similar to or higher than those of subjects classed as “active or highly active.” The diversity of factors and situations present in this type of research makes it difficult to establish an accurate system of categories and correlations.

## Strengths and Weaknesses

This research has a number of strengths and weaknesses which should be acknowledged and taken into account in future studies.

One of the strengths of the study is the use of accelerometers to obtain objective data regarding habitual PA among the study sample. Nevertheless, it is important to highlight the limitations associated with this type of device (Cain et al., 2013), such as its inability to measure certain types of PA (e.g., bicycle, cross-trainer, and strength training) and the need to remove it during high-intensity activities involving physical contact. As a result of these restrictions, recorded PA may sometimes be lower than the subject’s actual level of activity (Cuddihy et al., 2006), especially in the case of high-intensity activity.

One weakness associated with this strength is the limited size of the sample. A larger sample size would make it possible to draw more consistent conclusions from the data analysis, especially in relation to multivariate testing.

## Data Availability Statement

The datasets generated and analyzed during the current study are available from the corresponding author on reasonable request.

## Ethics Statement

No ethical review or approval was required for this study on human participants, in accordance with the local legislation and institutional requirements. As the participants were under 18 years of age, written informed consent was obtained from the parents or guardians of each participant, in accordance with the local legislation and institutional requirements. In accordance with the Helsinki Declaration World Medical Association (2013), all participants were informed in advance of the aims of the study and the voluntary nature of their participation, and assured of the confidentiality of their answers and personal information. The research was carried out in accordance with the University of A Coruña Code of Research Ethics (https://www.udc.es/export/sites/udc/investigacion/_galeria_down/hrs4r/Codigo-etico-ENG.pdf_2063069299.pdf).

## Author Contributions

RF-V and EP-R conceived the hypotheses for the research and analyzed the data. RF-V, LV-G, and MC-G were involved in data collection and wrote the manuscript with substantive input from EP-R. All authors contributed to the interpretation and discussion of the statistical analysis data, and read and approved the final manuscript.

## Conflict of Interest

The authors declare that the research was conducted in the absence of any commercial or financial relationships that could be construed as a potential conflict of interest.
